# Characterization and mitigation option of greenhouse gas emissions from lactating Holstein dairy cows in East China

**DOI:** 10.1186/s40104-022-00721-3

**Published:** 2022-06-30

**Authors:** Peng Jia, Yan Tu, Zhihao Liu, Qi Lai, Fadi Li, Lifeng Dong, Qiyu Diao

**Affiliations:** 1 State Key Laboratory of Grassland Agro-ecosystems, Key Laboratory of Grassland Livestock Industry Innovation, Ministry of Agriculture and Rural Affairs, Engineering Research Center of Grassland Industry, Ministry of Education, College of Pastoral Agriculture Science and Technology, Lanzhou University, Lanzhou, 730020 People’s Republic of China; 2 Institute of Feed Research, Chinese Academy of Agricultural Sciences/Sino-US Joint Lab on Nutrition and Metabolism of Ruminant, Beijing, 100081 People’s Republic of China; 3 College of Animal Science and Technology, Henan University of Science and Technology, Luoyang, 471003 People’s Republic of China; 4 College of Animal and Veterinary Sciences, Southwest Minzu University, Chengdu, 610041 People’s Republic of China

**Keywords:** Enteric methane emissions, GreenFeed system, Holstein dairy cows, Mitigation option, Production performance

## Abstract

**Background:**

This study investigated greenhouse gas (GHG) emission characteristics of lactating Holstein dairy cows in East China and provided a basis for formulating GHG emission reduction measures. GreenFeed system was used to measure the amount of methane (CH_4_) and carbon dioxide (CO_2_) emitted by the cows through respiration. Data from a commercial cow farm were used to observe the effects of parity, body weight, milk yield, and milk component yield on CH_4_ and CO_2_ emissions.

**Results:**

Mean herd responses throughout the study were as follows: 111 cows completed all experimental processes, while 42 cows were rejected because they were sick or had not visited the GreenFeed system 20 times. On average, lactating days of cows was 138 ± 19.04 d, metabolic weight was 136.5 ± 9.5 kg, parity was 2.8 ± 1.0, dry matter intake (DMI) was 23.1 ± 2.6 kg/d, and milk yield was 38.1 ± 6.9 kg/d. The GreenFeed system revealed that CH_4_ production (expressed in CO_2_ equivalent, CO_2_-eq) was found to be 8304 g/d, ${\mathrm{CH}}_{4\left({\mathrm{CO}}_2-\mathrm{eq}\right)}$/DMI was 359 g/kg, ${\mathrm{CH}}_{4\left({\mathrm{CO}}_2-\mathrm{eq}\right)}$/energy-corrected milk (ECM) was 229.5 g/kg, total CO_2_ production (CH_4_ production plus CO_2_ production) was 19,201 g/d, total CO_2_/DMI was 831 g/kg, and total CO_2_/ECM was 531 g/kg. The parity and metabolic weight of cows had no significant effect on total CO_2_ emissions (*P* > 0.05). Cows with high milk yield, milk fat yield, milk protein yield, and total milk solids yield produced more total CO_2_ (*P* < 0.05), but their total CO_2_ production per kg of ECM was low (*P* < 0.05). The total CO_2_/ECM of the medium and high milk yield groups was 17% and 27% lower than that of the low milk yield group, respectively.

**Conclusions:**

The parity and body condition had no effect on total CO_2_ emissions, while the total CO_2_/ECM was negatively correlated with milk yield, milk fat yield, milk protein yield, and total milk solids yield in lactating Holstein dairy cows. Measurement of total CO_2_ emissions of dairy cows in the Chinese production system will help establish regional or national GHG inventories and develop mitigation approaches to dairy production regimes.

**Supplementary Information:**

The online version contains supplementary material available at 10.1186/s40104-022-00721-3.

## Background

Climate change caused by greenhouse gas (GHG) is a huge environmental challenge to mankind [1]. Manufacturing, agriculture, and electricity sectors are the primary sources of GHG emissions, with agricultural emissions accounting for about 24% of total emissions [2]. Livestock is a prime anthropogenic source of methane (CH_4_) emissions from the agricultural sector, accounting for 18% of global GHG emissions [3], of which ruminant livestock is responsible for 93% of all livestock GHG emissions globally [4], and the dairy cows have the largest GHG emissions [5]. Carbon dioxide (CO_2_) and CH_4_ are the first two components of GHG, and CH_4_ has 25 times more warming power in the near term than CO_2_ [2]. To indicate their global-warming potential in the atmosphere, CH_4_ emissions are commonly quantified in CO_2_-eq units [6]. Furthermore, the residence time of CH_4_ in the atmosphere is 12.2 years, which is much lower than that of CO_2_ [7]. CH_4_ reduction is the fastest way to quickly mitigate climate change in the short term; therefore, attention should be paid to the ruminant industry, especially the dairy industry.

Meeting the demand for animal protein products has become a primary challenge for global food security as the world’s population continues to expand [8]. Ruminants are almost the sole source of milk for humans, providing 644 million tons per year of fat- and protein-corrected milk, of which dairy cows contribute to 80% [9]. However, the anaerobic fermentation of fiber feed in the rumen inevitably produces CH_4_ and affects the climate. In addition, the production of CH_4_ will cause a loss of 5–14% of the total energy intake of dairy cows [9]. Milk output is estimated to double by 2050 as the global population continues to grow [10]. As the consumer demand for dairy products is increasing, the expansion of the dairy industry aggravates the accumulation of GHG in the atmosphere contributing to global warming [11]. Therefore, there will be an urgent need to avoid the negative effects on the environment and save dietary energy by reducing CH_4_ emissions from dairy cows [12]. This necessitates the improvement in the efficiency of dairy cow production resource utilization reducing the GHG emissions to ensure sustainable and clean dairy cow production in the future. Despite the advances in research on GHG emissions from animal husbandry in Europe and the United States, there is a dearth of a GHG emission database in China.

China has raised awareness of the harmfulness of GHG and taken measures to address them [13]. China’s per capita milk consumption is far lower than the global level, and efforts to vigorously develop the dairy industry to meet living needs are still needed [14]. Consequently, mitigation strategies for China’s dairy industry need to be widely investigated. To reduce GHG emissions from dairy cows, we need to understand the specific characteristics of GHG emissions from these animals. GHG emissions from dairy cows are affected by factors such as the breed, environment, diet, and physiological stage. Understanding these emissions requires a significant amount of basic research to establish a database. China lacks a local dairy cow GHG emissions database, and before this experiment, neither did China have the latest animal CH_4_ emission detection equipment (GreenFeed system). Furthermore, China did not measure the gas emissions from a large herd of cows. The purpose of this study was to accurately determine the GHG emissions of lactating Holstein dairy cows under normal feeding conditions using the GreenFeed system and to calculate the relationship between GHG emissions and parity, body weight, milk yield, and milk component yield. This would lay the foundation for determining the CH_4_ and CO_2_ emissions of lactating Holstein dairy cows in East China, as well as facilitating further studies on the GHG emission characteristics of lactating Holstein dairy cows and locally applicable GHG emission reduction approaches in China.

## Materials and methods

### Animals, diets, and experimental design

China’s dairy cows are mainly concentrated in the north, and milk production in the North China Plain accounts for 25% of the total milk production in China [15]. Hence, the test data from farms in the North China Plain would be more representative. The experiment was conducted at the Yinxiangweiye International Third Farm, which is a part of the Yinxiangweiye Group Co., Ltd. within Cao County, Shandong Province (34°83 N, 115°54E).

The 153 healthy lactating Holstein dairy cows were selected from this farm as experimental cows, and these animals were housed in a barn. The parities of 153 lactating Holstein dairy cows ranged from 2 to 5, the days in milk from 104 to 182 d, and milk yield from 25.9 to 53.7 kg/d.

The cows were kept in a freestall barn (200 m × 10 m) and had free access to drinking water and saltlicks. The cows were fed a basal TMR with a forage:concentrate ratio of 40:60 on a dry matter (DM) basis, and the composition of the TMR was the same throughout the experiment (Table 1). The TMRs were provided three times daily in 4:3:3 proportions by an automatic feed wagon to guarantee ad libitum intake (aiming at 10% refusals), with feeding times of approximately 08:30, 15:30, and 23:30 h.

**Table 1:** Ingredient and chemical composition of the basal diet fed to lactating Holstein dairy cows

Items	Content, % of DM
Ingredient composition^a^	
Corn silage	21.10
Alfalfa hay	14.75
Oatgrass hay	3.46
Dandelion hay	0.69
Steam-flaked corn	14.77
Soybean meal	14.25
Corn flour	11.80
Beet pulp	5.11
Whole cottonseed	4.39
Rapeseed meal	2.56
Extruded soybean	1.64
Mineral-vitamin premix^b^	5.48
Calculated chemical composition	
OM	97.80
CP	16.78
EE	5.48
NDF	32.75
ADF	22.09
Ca	0.82
P	0.46
NE_L_, MJ/kg	7.46

^a^*DM* dry matter, *OM* organic matter, *CP* crude protein, *EE* ether extract, *NDF* neutral detergent fiber, *ADF* acid detergent fiber, *Ca* calcium, *P* phosphorus, *NE*_*L*_ net energy values were estimated based on NRC (2001)

^b^ The premix contained 140 g/kg of Mg, 122 g/kg of Ca, 93 g/kg of Na, 50 g/kg of K, 48 g/kg of Fe, 24 g/kg of P, 2 g/kg of S, 999 mg/kg of Zn, 580 mg/kg of Mn, 360 mg/kg of Cu, 180,070 IU of VA, 30,000 IU of VD and 601 IU of VE

The whole experiment was completed in 120 d with measurements of enteric CH_4_ and CO_2_ emissions, DMI, milk production, milk composition, and body weight of cows. There were four experimental periods, each 30 d long, and in experimental periods 1–4, 40, 40, 40, and 33 cows were randomly selected for measurement, respectively. The data of lactating Holstein dairy cows was divided into three groups according to parity, metabolic weight (MW), milk yield, milk fat yield, milk protein yield, and total milk solids yield. In addition, to be divided into three groups according to second parity (SP), third parity (TP), and fourth and above parity (FAP), the others were divided into three groups based on the standard deviation (SD): less than mean – 0.5 × SD, mean ± 0.5 × SD, and more than mean + 0.5 × SD. According to MW, milk yield, milk fat yield, milk protein yield, and total milk solids yield, the cows were divided into low metabolic weight (LMW), medium metabolic weight (MMW), and high metabolic weight (HMW) groups; low milk yield (LMY), medium milk yield (MMY), and high milk yield (HMY) groups; low milk fat yield (LMFY), medium milk fat yield (MMFY), and high milk fat yield (HMFY) groups; low milk protein yield (LMPY), medium milk protein yield (MMPY), and high milk protein yield (HMPY) groups; low total milk solids yield (LTMSY), medium total milk solids yield (MTMSY), and high total milk solids yield (HTMSY) groups, respectively.

### Measurement of methane and carbon dioxide emissions from lactating Holstein dairy cows using GreenFeed system

#### GreenFeed system

The GreenFeed system is the latest technique to directly measure the enteric greenhouse gas emissions and other gases (H_2_, O_2_) from animals. It is non-invasive, has a short measurement time and can be used in a large group of animals [7]. In the present study, it was necessary to ensure that the 153 cows were in a natural feeding state to obtain the actual gas emissions data of Chinese lactating Holstein dairy cows. Therefore, the GreenFeed system was the most suitable for the experiment.

#### Determination of methane and carbon dioxide emissions from lactating Holstein dairy cows

Respiratory gas exchange measurements were performed over the entire experimental period. Two GreenFeed units (C-Lock Inc., Rapid City, SD, USA) were permanently available for measuring gas emissions from cows according to the methods of Huhtanen et al. [16]. Before the measurements, the cows were allowed to adapt to the units for 5 d. Span gas (O_2_, CO_2_, and CH_4_) and zero gas (N_2_) calibrations were performed once a week. The standard gases consisted of two concentrations of O_2_ (2000 and 2100 ppm), 1500 ppm each of CO_2_ and CH_4_ for span gas, and 100% N_2_ (99.999% pure) for zero gas. A CO_2_ recovery test was conducted every 2 weeks during the entire experiment; the mean recovery was 100 ± 5%. Airflow was maintained above the manufacturer’s recommended rate of 26 L/s by cleaning the air filter when the flow rate approached this level. Alfalfa pellets (Ningxia Guyuan Forage Co., Ltd., Guyuan City, NX, CHN) were offered as bait feed to regularly entice the cows to visit the GreenFeed system. The weight of the alfalfa pellets obtained when each cow visited the units was recorded and used to calculate the DMI. The units were configured to allow each animal to visit at a minimum of 5-h intervals. During each visit, the cows were given eight drops of 30 g alfalfa pellets every 40 s, and the head position remained relatively stable for more than 3 min as a valid visit. More than 20 valid data points were ensured for each cow, and the average value was calculated as the final data; otherwise, it would be eliminated.

### Data and sample collection

#### Collection and analysis of the feed samples

During the entire experiment, the feed offered and refused were recorded daily for the barn to calculate the average feed intake of the cows. The TMR samples were collected once a week and were combined to obtain representative samples for the entire period of the experiment for analysis. The methods used were DM (Method 942.05; AOAC International, 1995) [17], CP (Method 990.03; AOAC International, 2000) [18], amylase-treated NDF (Van Soest et al., 1991) [19], EE (Method 2003.05; AOAC International, 2006) [20], ADF (Method 973.18; AOAC International, 2000) [18], ash (Method 942.05; AOAC International, 2000) [18], and minerals (Method 985.01; AOAC International, 2000) [18]. The GE content was determined using automatic oxygen bomb calorimetry (Parr Instrument Inc., Moline City, IL, USA). The ingredient and chemical composition of the basal diet were shown in Table 1.

#### Determination of body weight, milk yield, and milk composition of lactating Holstein dairy cows

Cows were weighed before morning feeding with an electronic loadometer (Zhengfeng Loadometer Co., Ltd., Shanghai, CHN) on the second day after completing the measurement of gas emissions. Cows were milked three times daily at 08:00, 15:00, and 23:00 h; milk yield was digitally logged with gravimetric milk recorders (Afimilk Co., Ltd., Kibbutzk, IL) at each milking. Milk samples were collected from three consecutive milkings on the second day after completing the measurement of gas emissions, and the collected milk samples were mixed in a ratio of 4:3:3. The samples (~ 50 mL) were preserved with 6% potassium dichromate (K_2_Cr_2_O_7_), stored at 4 °C, and analyzed within 3 d. Finally, the milk samples were submitted to the Shandong Province Testing Center for the analysis of milk fat, protein, and total milk solids. Fat-corrected milk (FCM 4%, kg/d) = 0.4 × milk yield (kg/d) + 15 × fat yield (kg/d) [21]. The energy-corrected milk (ECM, kg/d) = milk yield (kg/d) × [(38.3 × %fat × 10 + 24.2 × %protein × 10 + 16.54 × %lactose × 10 + 20.7) ÷ 3140] [22].

### Calculations

The conversion factor [2] was used to convert CH_4_ to CO_2_ equivalents. The total CO_2_ emissions were equal to the combined CH_4_ and CO_2_ exhaled by lactating Holstein dairy cows.

1 $$ {\mathrm{CH}}_4{\mathrm{CO}}_2\left(\mathrm{g}/\mathrm{d}\right)={\mathrm{CH}}_4\mathrm{emissions}\left(\mathrm{g}/\mathrm{d}\right)\times 25 $$

2 $$ \mathrm{Total}{\mathrm{CO}}_2\left(\mathrm{g}/\mathrm{d}\right)={\mathrm{CH}}_{4\left({\mathrm{CO}}_2-\mathrm{eq}\right)}\left(\mathrm{g}/\mathrm{d}\right)+{\mathrm{CO}}_2\mathrm{emissions}\left(\mathrm{g}/\mathrm{d}\right) $$

### Statistical analysis

A total of 153 lactating Holstein dairy cows were continuously adjusted according to management standards of this experimental farm based on hoof disease, mastitis, and other reasons. The 42 cows that made less than 20 valid visits to the system were eliminated, and 111 cows completed all data collection. All data were screened for normality using the UNIVARIATE procedure of SAS version 9.2 (SAS Institute Inc., Cary, NC, USA). The metabolic weight, milk yield, FCM yield, ECM yield, milk component (fat, protein, and total milk solids) percentage and yield, and GHG measurement, including total CO_2_, total CO_2_/MW, total CO_2_/ECM were analyzed using the one-way ANOVA procedure in SAS with repeated measures, according to the following model:

3 $$ {Y}_i=\mu +{T}_i+{e}_i $$

where *Y*_*i*_ is the dependent variable, *μ* is the overall mean, *T*_*i*_ is the effect of treatment (*i* = 1, 2, 3), and *e*_*i*_ is the residual error. The statistical significance was defined as *P* ≤ 0.05. Differences were considered to be a tendency toward significance at 0.05 < *P* ≤ 0.10.

## Results

### Carbon dioxide emissions of lactating Holstein dairy cows

The data for the overall herd are displayed in Table 2. The mean parity of the cows in the experiment was 2.8 ± 1.0, the mean lactation days was138 ± 19 d, the mean metabolic weight was 136.5 ± 9.5 kg, the mean DMI was 23.1 ± 2.6 kg/d, and the mean milk yield was 38.1 ± 6.9 kg/d. The CH_4_ emissions in the experiment were expressed in CO_2_ equivalent, and the CH_4_ production (8304 ± 1151 g/d), CH_4_ yield (359 ± 48 g/kg·DMI), and CH_4_ intensity (61.1 ± 9.3 g/kg·MW; 229.5 ± 48.1 g/kg·ECM) were calculated. The total CO_2_ production comprised the CH_4_ and CO_2_ production. The total CO_2_ production of the cows was 19,201 ± 2004 g/d, the total CO_2_ yield was 831 ± 84 g/kg·DMI, and the total CO_2_ intensity was 141 ± 17 g/kg·MW and 531 ± 103 g/kg·ECM.

**Table 2:** Feed intake, milk production and composition and carbon dioxide emission of lactating Holstein dairy cows (*n*^a^ = 111)

Items^b^	Mean	Minimum	Maximum	SD^c^
Animal description				
Age, months	51.7	36.3	89.9	12.7
Parity number	2.8	2.0	5.0	1.0
Days in milk, d	138	104	182	19
Metabolic weight, kg	136.5	116.4	160.1	9.5
Dry matter intake, kg/d	23.1	17.6	33.7	2.6
Milk production and composition				
Milk yield, kg/d	38.1	25.9	53.7	6.9
Feed efficiency, kg/kg	1.65	0.62	2.32	0.29
Milk fat yield, g/d	1414	545	2222	272
Milk protein yield, g/d	1247	446	1991	236
Total milk solids yield, g/d	4720	1811	6295	786
FCM yield, kg/d	36.4	29.8	54.8	6.6
ECM yield, kg/d	37.2	31	55.1	6.7
Greenhouse gas emissions				
${\mathrm{CH}}_{4\left({\mathrm{CO}}_2-\mathrm{eq}\right)}$, g/d	8304	5392	11,190	1151
${\mathrm{CH}}_{4\left({\mathrm{CO}}_2-\mathrm{eq}\right)}$/DMI, g/kg	359.4	227.1	492.6	48.3
${\mathrm{CH}}_{4\left({\mathrm{CO}}_2-\mathrm{eq}\right)}$/MW, g/kg	61.1	41.1	86.6	9.3
${\mathrm{CH}}_{4\left({\mathrm{CO}}_2-\mathrm{eq}\right)}$/ECM, g/kg	229.5	149.8	455.0	48.1
Total CO_2_, g/d	19,201	14,412	24,145	2004
Total CO_2_/DMI, g/kg	831.5	575.2	976.7	84.1
Total CO_2_/MW, g/kg	141.3	101.1	183.5	17.3
Total CO_2_/ECM, g/kg	531.1	343.3	1095.6	102.8

^a^*n*, number of observations in the data set

^b^Feed efficiency, milk yield ÷ dry matter intake (kg/kg). *FCM* Fat-corrected milk (kg/d) = 0.4 × milk yield (kg/d) + 15 × fat yield (kg/d). *ECM* Energy-corrected milk (kg/d) = milk yield (kg/d) × [(38.3 × fat (%) × 10 + 24.2 × protein (%) × 10 + 16.54 × lactose (%) × 10 + 20.7) ÷ 3140]. ${\mathrm{CH}}_{4\left({\mathrm{CO}}_2-\mathrm{eq}\right)}$, the CH_4_ emission in the experiment was expressed as CO_2_ equivalent, ${\mathrm{CH}}_{4\left({\mathrm{CO}}_2-\mathrm{eq}\right)}$ (g/d) = CH_4_ emissions (g/d) × 25. *DMI* dry matter intake (kg/d). *MW* metabolic weight (kg). Total CO_2_, total CO_2_ production (g/d) = ${\mathrm{CH}}_{4\left({\mathrm{CO}}_2-\mathrm{eq}\right)}$ (g/d) + CO_2_ production (g/d)

^c^*SD* Standard deviation

### Carbon dioxide emissions of lactating Holstein dairy cows with different parities

Using parity as the grouping standard, the cows were divided into SP, TP, and FAP groups (Table 3). There were no significant differences in the metabolic weight, milk production, milk compositions, and total CO_2_ emissions among the groups (*P* > 0.05). The milk yields of the SP, TP, and FAP groups were 38.5, 37.5, and 37.7 kg/d, and the total CO_2_ production was 19,236, 19,184, and 19,146 g/d, respectively.

**Table 3:** Carbon dioxide emissions of lactating Holstein dairy cows with different parities^a^

Items^b^	SP	TP	FAP	SEM	*P*-value
Animal description					
Metabolic weight, kg	133.5	140.5	138.3	2.31	0.633
Milk production and composition					
Milk yield, kg/d	38.5	37.5	37.7	4.43	0.379
Milk fat, %	3.71	3.70	3.83	0.27	0.737
Milk protein, %	3.26	3.27	3.37	0.35	0.645
Total milk solids, %	12.4	12.6	12.4	2.74	0.794
FCM yield, kg/d	36.8	35.8	36.5	4.33	0.594
ECM yield, kg/d	37.6	36.5	37.1	5.29	0.633
Carbon dioxide emissions					
Total CO_2_, g/d	19,236	19,184	19,146	19.03	0.291
Total CO_2_/MW, g/kg	144.5	137.2	139.0	4.78	0.516
Total CO_2_/ECM, g/kg	518.8	535.1	524.4	7.53	0.668

^a^*SP* second parity (*n* = 56), *TP* third parity (*n* = 30), *FAP* fourth and above parity (*n* = 25); *n*, number of observations in the data set

^b^*FCM* Fat-corrected milk (kg/d) = 0.4 × milk yield (kg/d) + 15 × fat yield (kg/d). *ECM* Energy-corrected milk (kg/d) = milk yield (kg/d) × [(38.3 × fat (%) × 10 + 24.2 × protein (%) × 10 + 16.54 × lactose (%) × 10 + 20.7) ÷ 3140]. Total CO_2_, total CO_2_ production (g/d) = ${\mathrm{CH}}_{4\left({\mathrm{CO}}_2-\mathrm{eq}\right)}$ (g/d) + CO_2_ production (g/d). *MW* metabolic weight (kg)

### Carbon dioxide emissions of lactating Holstein dairy cows with different metabolic weights

Table 4 shows the lactation performance and CO_2_ emissions of the cows with different metabolic weights. Compared with the LMW and MMW groups, the HMW group showed the trends of reducing milk, FCM, and ECM yields (0.05 < *P* < 0.1); the milk yield decreased by 1.9 and 1.88 kg/d in turns, and there was a tendency to increase the percentages of milk fat and milk protein (0.05 < *P* < 0.1). The total CO_2_ production of cows among the three groups was 18,996, 19,269, and 19,339 g/d (*P* > 0.05). The HMW group tended to decrease the total CO_2_/MW and increase the total CO_2_/ECM (0.05 < *P* < 0.1).

**Table 4:** Carbon dioxide emissions of lactating Holstein dairy cows with different metabolic weights^1^

Items^2^	LMW	MMW	HMW	SEM	*P*-value
Metabolic weight, kg	126.3^c^	136.4^b^	147.8^a^	2.89	0.026
Milk production and composition					
Milk yield, kg/d	38.7	38.5	36.8	2.02	0.057
Milk fat, %	3.66	3.73	3.81	0.26	0.062
Milk protein, %	3.22	3.28	3.37	0.29	0.059
Total milk solids, %	12.4	12.3	12.7	1.03	0.826
FCM yield, kg/d	36.7	36.9	35.6	2.34	0.068
ECM yield, kg/d	37.4	37.5	36.5	2.62	0.084
Carbon dioxide emissions					
Total CO_2_, g/d	18,996	19,269	19,339	20.83	0.475
Total CO_2_/MW, g/kg	150.6	141.3	131.1	4.03	0.063
Total CO_2_/ECM, g/kg	517.6	530.6	546.6	9.13	0.078

^1^*LMW* low metabolic weight (< 131.7, *n* = 36), *MMW* medium metabolic weight (131.7–141.2, *n* = 42), *HMW* high metabolic weight (> 141.2, *n* = 33), *n* number of observations in the data set

^2^*FCM* Fat-corrected milk (kg/d) = 0.4 × milk yield (kg/d) + 15 × fat yield (kg/d). *ECM* Energy-corrected milk (kg/d) = milk yield (kg/d) × [(38.3 × fat (%) × 10 + 24.2 × protein (%) × 10 + 16.54 × lactose (%) × 10 + 20.7) ÷ 3140. Total CO_2_, total CO_2_ production (g/d) = ${\mathrm{CH}}_{4\left({\mathrm{CO}}_2-\mathrm{eq}\right)}$ (g/d) + CO_2_ production (g/d). *MW* metabolic weight (kg)

^a-c ^Means in the same row with different superscripts are significantly different (*P* < 0.05)

### Carbon dioxide emissions of lactating Holstein dairy cows with different milk yields

**Fig. 1:**
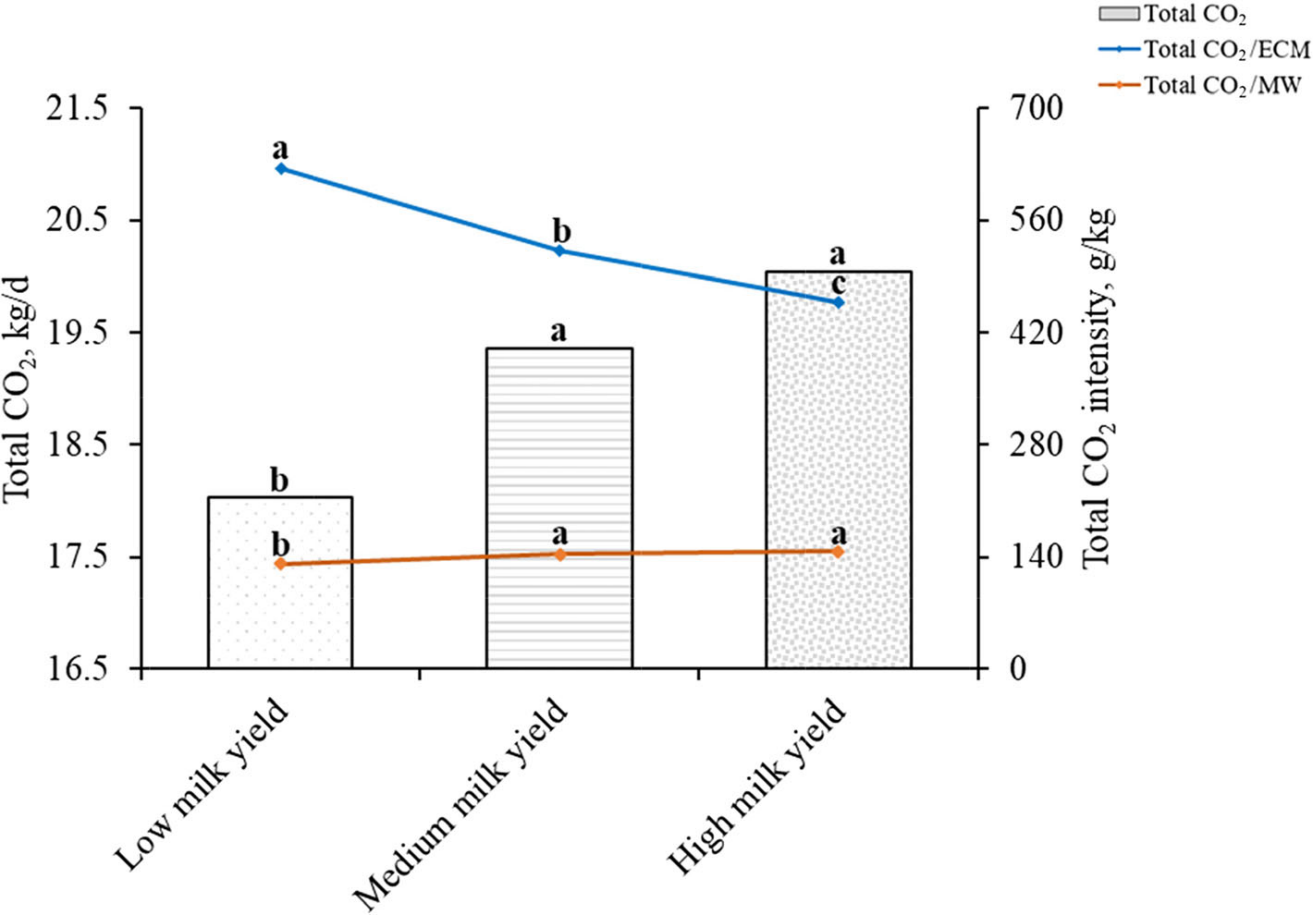
Carbon dioxide emissions of lactating Holstein dairy cows with different milk yields. Total CO_2_: *P* < 0.05, Total CO_2_/MW: *P* < 0.05, Total CO_2_/ECM: *P* < 0.05. MW: metabolic weight (kg). Total CO_2_, total CO_2_ production (g/d) = ${\mathrm{CH}}_{4\left({\mathrm{CO}}_2-\mathrm{eq}\right)}$ (g/d) + CO_2_ production (g/d). ECM, Energy-corrected milk (kg/d) = milk yield (kg/d) × [(38.3 × fat (%) × 10 + 24.2 × protein (%) × 10 + 16.54 × lactose (%) × 10 + 20.7) ÷ 3140]

The milk production, milk composition, and CO_2_ emissions of cows with different milk yields are shown in Table 5, Fig. 1, and Table S1. Milk fat, milk protein, and total milk solids percentages of cows in the HMY group were significantly lower than those in the LMY group (*P* < 0.05). FCM and ECM yields were proportional to the milk yield of the cows (*P* < 0.05). The total CO_2_/MW of cows in the MMY and HMY groups were significantly higher than those in the LMY group (*P* < 0.05). Milk yield of cows had a significant positive relationship with total CO_2_ production and a negative relationship with total CO_2_/ECM (*P* < 0.05). The total CO_2_ production of the three groups was 18,033, 19,364, and 20,048 g/d, with the HMY group was 11% higher than the LMY group; however, the total CO_2_/ECM of the LMY group was 36% higher than that of the HMY group.

**Table 5:** Lactation performance of lactating Holstein dairy cows with different milk yields^1^

Items^2^	LMY	MMY	HMY	SEM	*P*-value
Milk yield, kg/d	29.6^c^	38.2^b^	45.8^a^	2.08	0.024
Milk fat, %	3.88^a^	3.70^b^	3.65^b^	0.31	0.038
Milk protein, %	3.36^a^	3.30^ab^	3.21^b^	0.27	0.031
Total milk solids, %	13.0^a^	12.5^ab^	12.0^b^	0.99	0.040
FCM yield, kg/d	28.9^c^	36.4^b^	43.5^a^	2.38	0.042
ECM yield, kg/d	29.6^c^	37.3^b^	44.2^a^	2.48	0.039

^1^*LMY* low milk yield (< 34.7, *n* = 30), *MMY* medium milk yield (34.7–41.5, *n* = 49), *HMY* high milk yield (> 41.5, *n* = 32), *n* number of observations in the data set

^2^*FCM* Fat-corrected milk (kg/d) = 0.4 × milk yield (kg/d) + 15 × fat yield (kg/d). *ECM* Energy-corrected milk (kg/d) = milk yield (kg/d) × [(38.3 × fat (%) × 10 + 24.2 × protein (%) × 10 + 16.54 × lactose (%) × 10 + 20.7) ÷ 3140]

^a-c^Means in the same row with different superscripts are significantly different (*P* < 0.05)

### Carbon dioxide emissions of lactating Holstein dairy cows with different milk fat yields

**Fig. 2:**
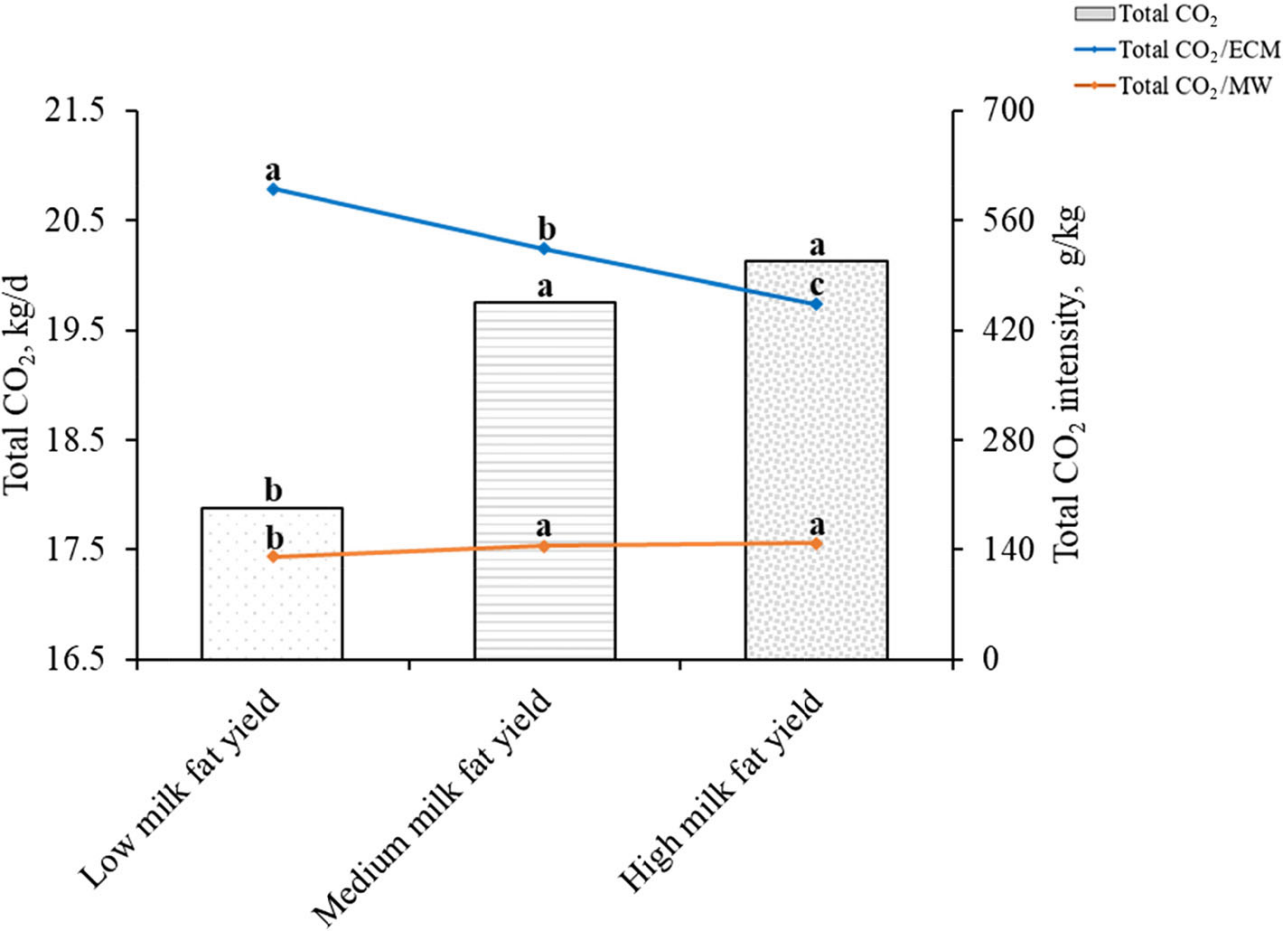
Carbon dioxide emissions of lactating Holstein dairy cows with different milk fat yields. Total CO_2_: *P* < 0.05, Total CO_2_/MW: *P* < 0.05, Total CO_2_/ECM: *P* < 0.05. MW: metabolic weight (kg). Total CO_2_, total CO_2_ production (g/d) = ${\mathrm{CH}}_{4\left({\mathrm{CO}}_2-\mathrm{eq}\right)}$ (g/d) + CO_2_ production (g/d). ECM, Energy-corrected milk (kg/d) = milk yield (kg/d) × [(38.3 × fat (%) × 10 + 24.2 × protein (%) × 10 + 16.54 × lactose (%) × 10 + 20.7) ÷ 3140]

The cows were divided into LMFY, MMFY, and HMFY groups (Table 6, Fig. 2, and Table S2). There were significant differences in the milk yield, FCM yield, ECM yield, milk fat percentage, and milk protein percentage (*P* < 0.05), which were positively correlated with milk fat yield. However, there was no difference in the total milk solids percentage among the groups (*P* > 0.05). The total CO_2_ and total CO_2_/MW of the MMFY and HMFY groups were significantly higher than those of the LMFY group (*P* < 0.05), but there was no difference between the MMFY and HMFY groups (*P* > 0.05). The total CO_2_ levels of the three groups were 17,884, 19,751, and 20,132 g/d, respectively. There were significant differences in total CO_2_/ECM among the three groups (*P* < 0.05), total CO_2_/ECM decreased with increasing milk fat yield. The total CO_2_/ECM of the MMFY and HMFY groups was 76.1 g/kg and 146.7 g/kg lower than that of the LMFY group, respectively.

**Table 6:** Lactation performance of lactating Holstein dairy cows with different milk fat yields^1^

Items^2^	LMFY	MMFY	HMFY	SEM	*P*-value
Milk fat yield, g/d	1138^c^	1435^b^	1732^a^	15.38	0.029
Milk yield, kg/d	32.1^c^	39.1^b^	44.2^a^	2.01	0.038
Milk fat, %	3.59^c^	3.71^b^	3.94^a^	0.27	0.041
Milk protein, %	3.18^c^	3.31^b^	3.41^a^	0.32	0.048
Total milk solids, %	12.6	12.3	12.6	0.93	0.863
FCM yield, kg/d	29.9^c^	37.2^b^	43.6^a^	2.79	0.036
ECM yield, kg/d	30.5^c^	37.8^b^	44.7^a^	2.95	0.041

^1^*LMFY* low milk fat yield (< 1278, *n* = 39), *MMFY* medium milk fat yield (1278–1550, *n* = 41), *HMFY* high milk fat yield (> 1550, *n* = 31), *n* number of observations in the data set

^2^*FCM* Fat-corrected milk (kg/d) = 0.4 × milk yield (kg/d) + 15 × fat yield (kg/d). *ECM* Energy-corrected milk (kg/d) = milk yield (kg/d) × [(38.3 × fat (%) × 10 + 24.2 × protein (%) × 10 + 16.54 × lactose (%) × 10 + 20.7) ÷ 3140]

^a-c^Means in the same row with different superscripts are significantly different (*P* < 0.05)

### Carbon dioxide emissions of lactating Holstein dairy cows with different milk protein yields

**Fig. 3:**
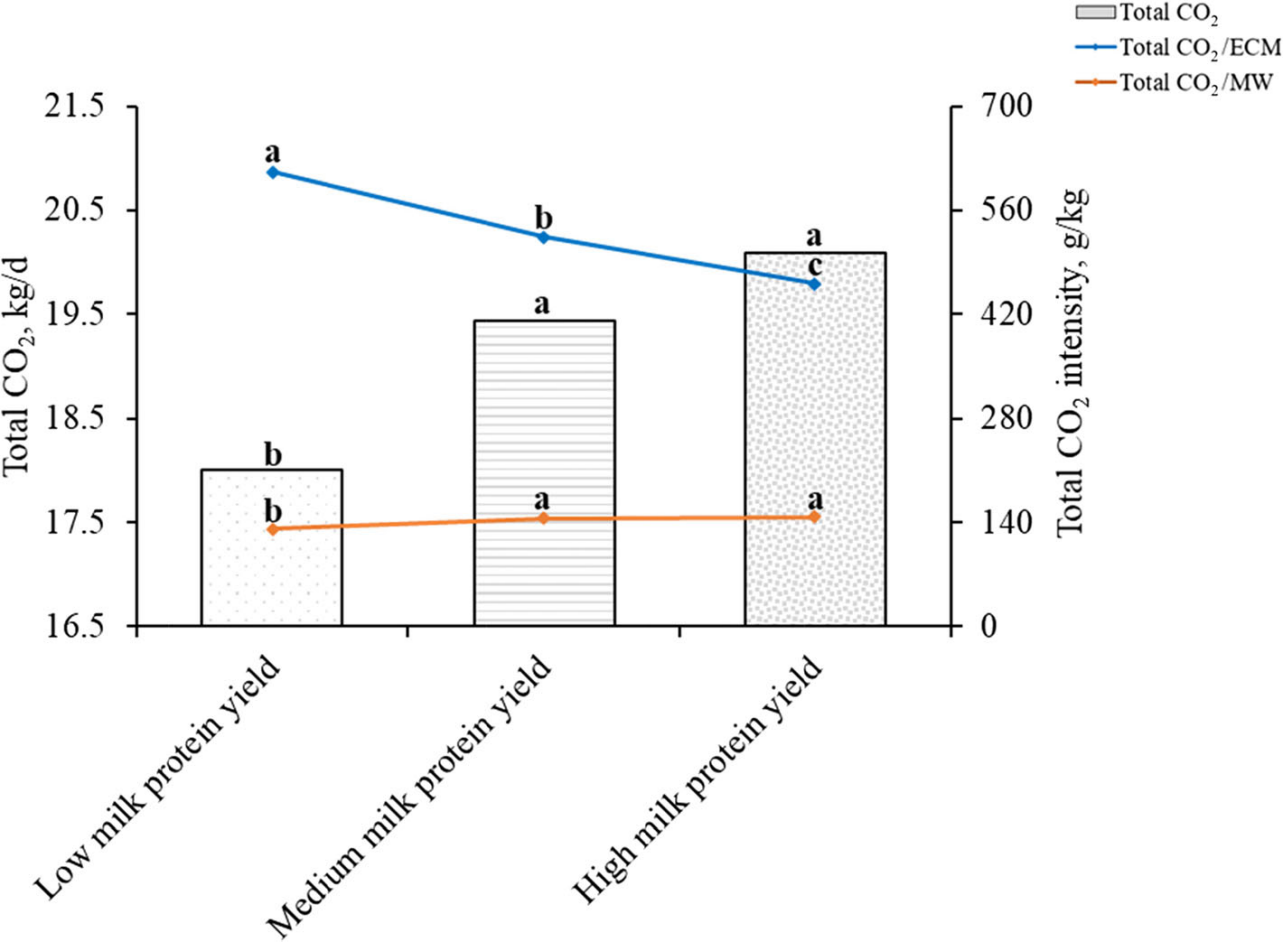
Carbon dioxide emissions of lactating Holstein dairy cows with different milk protein yields. Total CO_2_: *P* < 0.05, Total CO_2_/MW: *P* < 0.05, Total CO_2_/ECM: *P* < 0.05. MW: metabolic weight (kg). Total CO_2_, total CO_2_ production (g/d) = ${\mathrm{CH}}_{4\left({\mathrm{CO}}_2-\mathrm{eq}\right)}$ (g/d) + CO_2_ production (g/d). ECM, Energy-corrected milk (kg/d) = milk yield (kg/d) × [(38.3 × fat (%) × 10 + 24.2 × protein (%) × 10 + 16.54 × lactose (%) × 10 + 20.7) ÷ 3140]

The cows were separated into three groups based on the milk protein yield (Table 7, Fig. 3, and Table S3). There were significant differences in the milk yield, FCM yield, ECM yield, and milk fat percentage (*P* < 0.05); these values increased with an increase in the milk protein yield. The milk protein percentages of MMPY and HMPY groups were significantly higher than that of the LMPY group (*P* < 0.05), but there was no difference between MMPY and HMPY groups (*P* > 0.05). In addition, there was no difference in the total milk solids percentage among the three groups (*P* > 0.05). The total CO_2_ and total CO_2_/MW of the MMPY and HMPY groups were significantly higher than those of the LMPY group (*P* < 0.05). The total CO_2_/ECM of the three groups was 612.2, 524.4, and 461.5 g/kg, respectively. There were significant differences in the total CO_2_/ECM among the three groups (*P* < 0.05), the LMPY group was the highest, the HMPY group was the lowest, and the MMPY group was in the middle.

**Table 7:** Lactation performance of lactating Holstein dairy cows with different milk protein yields^1^

Items^2^	LMPY	MMPY	HMPY	SEM	*P*-value
Milk protein yield, g/d	983^c^	1254^b^	1490^a^	16.7	0.024
Milk yield, kg/d	31.6^c^	38.1^b^	44.2^a^	2.39	0.041
Milk fat, %	3.65^c^	3.71^b^	3.83^a^	0.31	0.039
Milk protein, %	3.16^b^	3.31^ab^	3.39^a^	0.28	0.031
Total milk solids, %	11.8	12.5	12.3	0.79	0.762
FCM yield, kg/d	29.7^c^	36.3^b^	42.9^a^	2.98	0.039
ECM yield, kg/d	30.1^c^	37.1^b^	43.9^a^	3.65	0.025

^1^*LMPY* low milk protein yield (< 1130, *n* = 35), *MMPY* medium milk protein yield (1130–1364, *n* = 39), *HMPY* high milk protein yield (> 1364, *n* = 37), *n* number of observations in the data set

^2^*FCM* Fat-corrected milk (kg/d) = 0.4 × milk yield (kg/d) + 15 × fat yield (kg/d). *ECM* Energy-corrected milk (kg/d) = milk yield (kg/d) × [(38.3 × fat (%) × 10 + 24.2 × protein (%) × 10 + 16.54 × lactose (%) × 10 + 20.7) ÷ 3140]

^a-c^Means in the same row with different superscripts are significantly different (*P* < 0.05)

### Carbon dioxide emissions of lactating Holstein dairy cows with different total milk solids yields

**Fig. 4:**
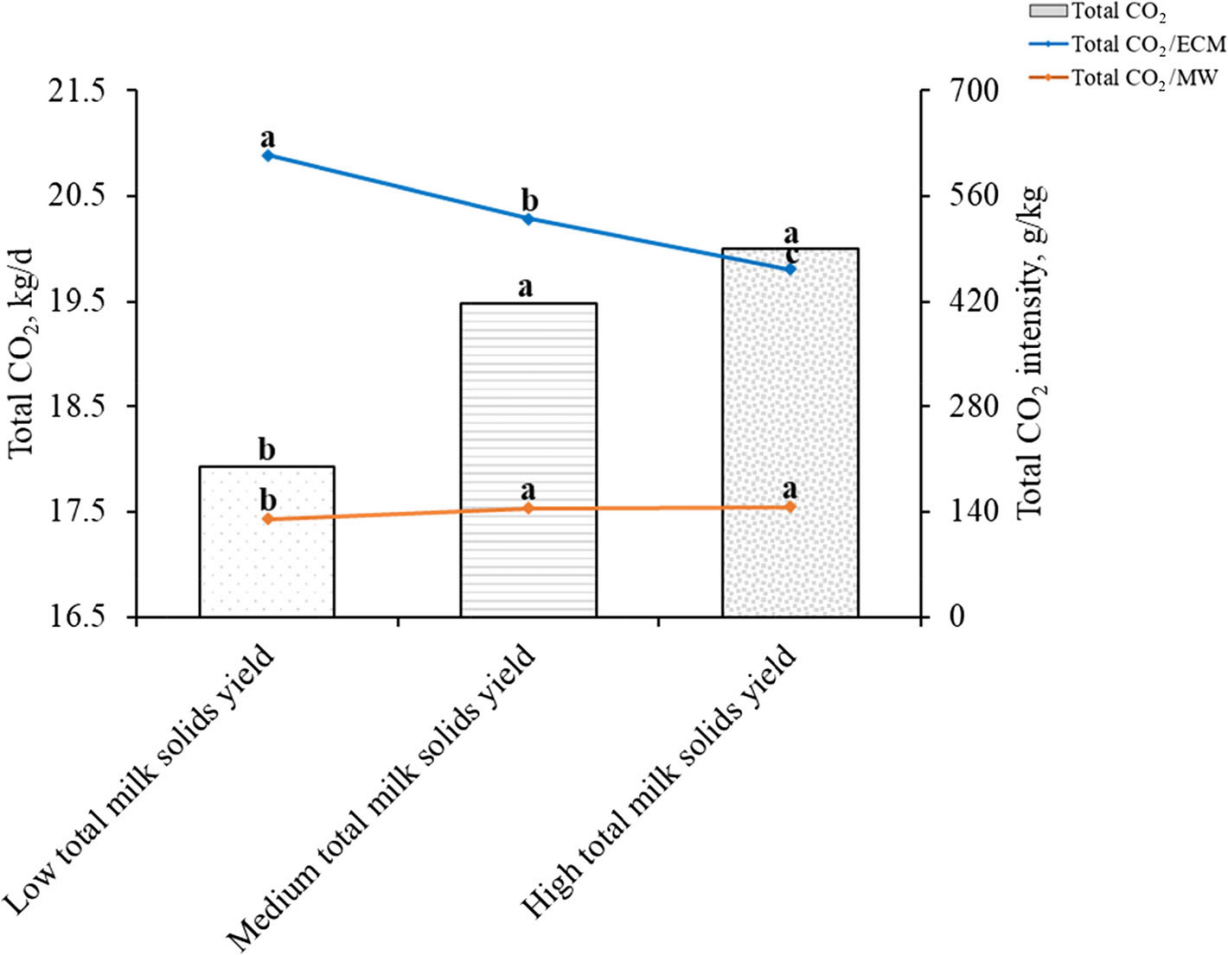
Carbon dioxide emissions of lactating Holstein dairy cows with different total milk solids yields. Total CO_2_: *P* < 0.05, Total CO_2_/MW: *P* < 0.05, Total CO_2_/ECM: *P* < 0.05. MW: metabolic weight (kg). Total CO_2_, total CO_2_ production (g/d) = ${\mathrm{CH}}_{4\left({\mathrm{CO}}_2-\mathrm{eq}\right)}$ (g/d) + CO_2_ production (g/d). ECM, Energy-corrected milk (kg/d) = milk yield (kg/d) × [(38.3 × fat (%) × 10 + 24.2 × protein (%) × 10 + 16.54 × lactose (%) × 10 + 20.7) ÷ 3140]

Milk, FCM, and ECM yields increased with the increase of total milk solids yield, and there were significant differences among the three groups (*P* < 0.05, Table 8). However, there were no significant differences in the milk fat, milk protein, and total milk solids percentages among the three groups (*P* > 0.05). There were significant differences in the total CO_2_ production and total CO_2_/ECM of cows between each group (*P* < 0.05), and total milk solids had a positive relationship with total CO_2_ production and a negative relationship with the total CO_2_/ECM (Fig. 4, and Table S4). The total CO_2_ production of each group was 17,924, 19,486, and 19,998 g/d, and the total CO_2_/ECM of the MTMSY and HTMSY groups was 14% and 25% lower than that of the LTMSY group, respectively. The total CO_2_/MW of cows in the MTMSY and HTMSY groups was significantly higher than that in the LTMSY group (*P* < 0.05), and there was no difference between the MTMSY and HTMSY groups (*P* > 0.05).

**Table 8:** Lactation performance of lactating Holstein dairy cows with different total milk solids yields^1^

Items^2^	LTMSY	MTMSY	HTMSY	SEM	*P*-value
Total milk solid yield, g/d	3789^c^	4714^b^	5512^a^	20.3	0.031
Milk yield, kg/d	31.0^c^	37.6^b^	44.5^a^	2.36	0.021
Milk fat, %	3.74	3.73	3.73	0.20	0.916
Milk protein, %	3.27	3.31	3.29	0.22	0.871
Total milk solids, %	12.4	12.6	12.4	0.86	0.893
FCM yield, kg/d	29.5^c^	36.0^b^	42.7^a^	2.34	0.036
ECM yield, kg/d	29.9^c^	36.9^b^	43.6^a^	2.35	0.041

^1^*LTMSY* low total milk solids yield (< 4325, *n* = 33); *MTMSY* medium total milk solids yield (4325–5115, *n* = 39); *HTMSY* high total milk solids yield (> 5115, *n* = 39); *n*, number of observations in the data set

^2^*FCM* Fat-corrected milk (kg/d) = 0.4 × milk yield (kg/d) + 15 × fat yield (kg/d). *ECM* Energy-corrected milk (kg/d) = milk yield (kg/d) × [(38.3 × fat (%) × 10 + 24.2 × protein (%) × 10 + 16.54 × lactose (%) × 10 + 20.7) ÷ 3140]

^a-c^Means in the same row with different superscripts are significantly different (*P* < 0.05)

## Discussion

### Carbon dioxide emissions of lactating Holstein dairy cows

The data of this experiment were obtained under the normal feeding conditions of the dairy farm, so it had stronger reliability and representativeness [23]. This analysis contributed to further understanding of the GHG emission characteristics of Chinese lactating Holstein dairy cows.

In terms of GHG emissions, the CH_4_ production was 8304 g/d (expressed as CO_2_ equivalents), CH_4_ yield was 359 g/kg·DMI (CO_2−_eq), CH_4_ intensity was 229.5 g/kg·ECM (CO_2−_eq), and total CO_2_ production was 19,201 g/d. Niu et al. [24] summarized 2566 data points from Europe, the United States, and Australia. The CH_4_ production of dairy cows was 9225 g/d (CO_2−_eq), CH_4_ yield was 502.5 g/kg·DMI (CO_2−_eq), and CH_4_ intensity was 337.5 g/kg·ECM (CO_2−_eq). The results of Niu et al. [24] were higher than the results of the present experiment, probably because the Chinese Holstein lactating dairy cow diets were relatively higher in the concentrate-to-forage ratio, such as lower NDF (32.8% vs. 35.4%) and higher EE (5.5% vs. 3.5%) content decreased CH_4_ emissions [24–26]. Or the CH_4_ measurement method and the characteristics of the cows were different, Niu’s data were derived from Holstein, Ayrshire, Jersey, Brown Swiss, Simmental, and crossbred dairy cows measured using respiration chambers, the GreenFeed system, and sulfur hexafluoride (SF_6_) tracer technique [24]. Therefore, a test with the same measurement methods and similar cow characteristics was carried out. The lactating Holstein dairy cows had an average milk yield of 39.8 kg/d, DMI of 25.3 kg/d, DIM of 115 d, and body weight of 624 kg at the beginning of the experiment [27]. The results of Oh et al. [27] were similar to those of the present study, showing that the CH_4_ production of cows was 8425 g/d (CO_2−_eq), CH_4_ yield was 332.5 g/kg·DMI (CO_2−_eq), CH_4_ intensity was 231.5 g/kg·ECM (CO_2−_eq), and total CO_2_ production was 20,644 g/d. The present experiment showed that each Holstein lactating dairy cow emitted 7008 kg of the total CO_2_ per year in East China.

DMI is the primary factor affecting the emission of CH_4_ from the cows [7]. As a result, utilizing DMI to predict CH_4_ emissions is more accurate, but the data are more difficult to obtain. In addition, the different types of diets also have an impact on CH_4_ emissions. Therefore, the present study focused on Holstein lactating dairy cow’s variables, such as parity, weight, milk production, and milk composition; and their impact on exhaled GHG emissions was studied.

### Carbon dioxide emissions of lactating Holstein dairy cows with different parities

Parity is an essential physiological indicator in cows. The weight of the primiparous cows was lower, and the nutrients ingested by them were distributed to the body for growth, causing the metabolism, DMI, milk production, and fertility of primiparous cows to be different from those of the multiparous cows [28–30]. Only multiparous lactating Holstein dairy cows were chosen as experimental animals to eliminate this influencing factor. The present experiment showed that there was no difference in the metabolic body weight among lactating Holstein dairy cows of various parities, indicating that the physiological structure of cows matured after the second parity. In addition, there were no differences in the milk yield or milk component concentration among the different groups. Similar results have been reported in other studies. The milk yield of first parity was the lowest, but there were no differences among the second, third, and fourth parities [31]. The parity number also did not affect the contents of milk fat and protein during early lactation [31]. It is generally believed that after a cow reached a certain age, the lactation performance would decrease with the increase in parity [32]. This problem did not appear in the present experiment probably because of the proper daily feeding on the dairy farm, proper management of the herd, and the small number of cows with more than fourth parity.

Grandl et al. [32] showed that the CH_4_ emissions of the cows peaked during the second to third lactation period until CH_4_ production, CH_4_ yield, and CH_4_ intensity were low at about 6.5 years of age. There were no significant differences in the total CO_2_ production, total CO_2_/MW, and total CO_2_/ECM among the groups in this experiment. Chewing efficiency resulted in fiber degradation, which was the greatest of medium-aged cows [33]. Methane emissions had the concomitant relationship with fiber digestibility, so lower in young and old cows. Only a few cows over 6.5 years old were included in the present experiment, perhaps because it is very common to eliminate older cows in pursuit of higher feeding efficiency in Chinese dairy farms. Therefore, parity was not a factor affecting CO_2_ emissions from lactating Holstein dairy cows in the present experiment.

### Carbon dioxide emissions of lactating Holstein dairy cows with different metabolic weights

Contrary to the results of this experiment, it is generally believed that although the relationship between body weight and milk production is not very strong, the cows with high milk production tend to be larger [34]. Previous studies have shown that the body condition score directly affected by the body weight was negatively correlated with milk production, and negatively correlated with reproductive performance [35, 36]. The cow body weight seemed to be positively correlated with the incidence of metritis and milk somatic cell score [37, 38]. Therefore, excessive metabolic body weight would be detrimental to the lactation performance of lactating Holstein dairy cows.

According to Blaxter and Czerkawski [39], reducing CH_4_ production from the rumen provides more metabolizable energy utilization for the growth of body tissues. Hristov et al. [40] showed that the reduction in CH_4_ emission from Holstein cows significantly increased the rate of weight gain. Previous studies illustrated how a reduction in dietary GE loss, such as CH_4_, can increase the energy available for production purposes, that is, improve lactose and protein synthesis in milk, or restore weight loss during early lactation [40]. Although there was no significant difference in total CO_2_ production between cows with different metabolic weights, the total CO_2_ production of low metabolic weight cows were quantitatively lower than that of high metabolic weight cows in current experiment. Van Zijderveld et al. [41] concluded that weight gain did not always improve when the CH_4_ production in dairy cows was suppressed. For example, if the cows’ weight loss in early lactation has been restored, then the weight of middle lactation cows would remain stable.

Some studies have reported a negligible relationship between live weight and CH_4_ emissions, but lighter animals ate less and therefore produced less total gas emissions [42]. In contrast to these studies, there was no difference in the total CO_2_ production among the different groups in the present test. The CH_4_ emissions of dairy cows and the digestibility of dietary fiber showed similar changes, according to Grandl et al. [32]. It was speculated that although the cows with high metabolic weight had high feed intake, their dietary digestibility was low, therefore they did not affect enteric gas emissions. There was no doubt that the higher the metabolic weight, the lower the total CO_2_/MW. The numerical order of the total CO_2_/MW was HMW group < MMW group < LMW group. The milk yield of cows did not increase with an increase in the metabolic weight in this study. Therefore, the order of the size of the total CO_2_/ECM was the opposite to that of the total CO_2_/MW. Higher-weight cows had a negative impact on lactation performance and GHG reduction.

### Carbon dioxide emissions of lactating Holstein dairy cows with different milk yields

Bedö et al. [43] showed that milk component percentage was negatively correlated with milk yield of dairy cows. This is easy to understand: the higher the milk production, the lower the concentration of milk components [44]. The present experiment also demonstrated that the proportions of milk fat, milk protein, and total milk solids decreased with the increase of milk production.

Reducing GHG emissions is one of the key goals of dairy industry [12]. A previous study demonstrated a significant positive correlation between milk yields and CH_4_ emissions [45]. Gerber et al. [46] showed that higher milk yields result in higher CO_2_, CH_4_ and nitrous oxide emissions per cow. The result is in line with previous studies, which the total CO_2_ production of the HMY group was significantly higher than that of the LMY group by 2015 g/d in the current study. From the perspective of total CO_2_ production, the dairy cows with high milk yield did not seem to be conducive to the mitigation of total CO_2_. However, emissions per unit of animal products reflect the accuracy of management practices on the composite of feed intake, GHG emissions, and animal productivity [47]. Evaluating the total CO_2_ emission capacity of lactating Holstein dairy cows should be based on the CO_2_ production relative to ECM because the ultimate goal of the dairy farming industry was to obtain milk.

It is estimated that the rapid growth of the global population, combined with the improvements in global living standards, would lead to a 48% increase in global demand for dairy products between 2005 and 2050 [48]. As per the goal of the Chinese government’s dairy industry development, China is estimated to produce 45 million tons of milk by 2025, showing an increase of 40% over 2019 [49]. This would expand the dairy industry and increase the number of dairy cows. Although China is the world’s third-largest milk producer, low-productivity milk production has a greater impact on the environment compared to that from developed countries [50, 51]. The present experiment showed that the total CO_2_/ECM of the HMY group was significantly lower by 167 g/kg than that of the LMY group. In other words, the higher the milk yield of lactating Holstein dairy cows, the lower would be the total CO_2_ production per unit of ECM. Similarly, the study discovered that as milk production increases, GHG emissions per kg fat and protein corrected milk decrease significantly [46]. In 2019, the average milk production of dairy cows in China was only 5647 kg/head, which is lower than that of Europe and New Zealand, and there is still much room for improvement in milk production [56]. Therefore, development goals should be formulated for the dairy industry, by increasing the milk production of lactating Holstein dairy cows. It is possible to feed fewer cows to obtain more milk while reduce GHG emissions.

### Carbon dioxide emissions of lactating Holstein dairy cows with different milk component yields

Milk fat percentage is not only an important index for evaluating milk quality but also for evaluating the lactation performance and mammary gland health of dairy cows. There were significant differences in the milk production and milk component percentage between the different milk fat yield groups, and that of the HMFY group was higher than that of the LMFY group, except for the total milk solids percentage. Generally, the concentration of milk component decreased with the increase of milk yield due to dilution effect [52]. However, the results of this experiment revealed that milk fat yield was higher only when the milk yield and milk fat percentage were both high. The reason for there being no differences between the total milk solids percentages might be because the milk fat and the milk protein percentages were positively correlated, while the lactose percentage was negatively correlated with them, which ultimately balanced the total milk solids percentage among the groups [53].

The total CO_2_ production in the HMFY and MMFY groups was higher than that in the LMFY group. This is probably because a higher milk yield would require higher feed intake, digestion, absorption, and metabolism, and DMI is a major driver of enteric CH_4_ emission [7], which would in turn produce more CH_4_ and CO_2_ [7]. There was no difference between the HMFY and MMFY groups, indicating that the digestive and metabolic functions of the animals had an upper limit and could not continue to increase. The present study has concluded that the weight of dairy cows did not increase because of the increase in milk yield, so the total CO_2_/MW of MMFY and HMFY groups was significantly higher than that of the LMFY group. However, the total CO_2_/ECM of cows with a high milk fat yield was lower than that of cows with low milk fat yield. The total CO_2_/ECM of the MMFY and HMFY groups was 13% and 24% lower than that of the LMFY group. From the perspective of animal products, the cows with higher milk fat yield are more conducive to reducing GHG emissions.

Protein is an important nutrient component of milk that can provide people with high-quality functional proteins, and its yield is closely related to economic benefits. We have been trying to improve the milk protein yield of lactating Holstein dairy cows through herd management, nutrition, and genetics [8]. There were significant differences in the milk yield, milk fat and milk protein percentages among the groups, and the HMPY group was higher than the LMFY group. The present experiment showed that milk protein percentage decreased with the increase of milk yield. However, it can be seen from the data of the cows with various milk protein yields that the milk protein yield was higher only when the milk yield and milk protein percentage were both high. These results were consistent with those reported by Xue et al. [8]. In this experiment, the total CO_2_ production and total CO_2_/MW of cows with higher milk protein yield were higher than those of cows with lower milk protein yield. However, the GHG emissions of cows with higher milk protein yield were lower than those of cows with lower milk protein yield, when the emissions expressed as per kg of ECM. The total CO_2_/ECM of the MMPY and HMPY groups was 87.8 and 150.7 g/kg lower than that of the LMPY group, respectively. The rationale for this difference was the same as the difference in milk fat yield groups, and the cows with higher milk protein yields are more conducive to reducing GHG emissions.

In addition to the two major nutrients of milk fat and milk protein, milk also contains lactose, vitamins, and minerals; therefore, total milk solids is also an important indicator of milk quality. Milk yield was positively correlated with total milk solids yield, on the other hand, the concentration of milk component in the three groups did not differ in the current study. Cows with higher total milk solids yield had higher total CO_2_ production and total CO_2_/MW than these with lower total milk solids yield. However, the total CO_2_/ECM decreased with the increase of total milk solids yield of dairy cows. The number of cows and heifers required for the same total milk solids yield under different lactation performance conditions varied greatly [54]. In the dairy industry, the total milk solids yield is positively correlated with CH_4_ emissions [55], while CH_4_ intensity (per kg of milk production) decreases as milk yield improves [54]. In line with these earlier studies, with the increase in total milk solids yield of lactating Holstein dairy cows, the total CO_2_ intensity (CO_2_ per kg of ECM yield) decreased in this test. The total CO_2_/ ECM of LTMSY, MTMSY, and HTMSY groups was 614.3, 530.0, and 461.9 g/kg, respectively. However, with the improvement of the standards of living of the people of China, the development of the milk industry would be promoted. Therefore, it is important to determine the methods and strategies to find a balance between minimizing environmental impact and increasing animal productivity to meet the demands of the world population for animal protein. It is an effective carbon emission reduction measure to reduce the number of cows and the total CO_2_ intensity by increasing the milk component yield of lactating Holstein dairy cows.

## Conclusions

This study demonstrates that total CO_2_ emissions from lactating Holstein dairy cows in East China averaged 19,201 ± 2004 g/d, 831 ± 84 g/kg·DMI, 141 ± 17 g/kg·BW, and 531.1 ± 103 g/kg·ECM, respectively. Lactating Holstein dairy cows with low milk yield, milk fat yield, milk protein yield, and total milk solids yield produced less total CO_2_, but their total CO_2_ production per kg of ECM was higher. Therefore, it was concluded that selecting lactating Holstein dairy cows with less total CO_2_ production would probably reduce production efficiency and significantly increase the production cost of the dairy products. Low total CO_2_ intensity (total CO_2_/ECM) cows demonstrated higher efficiency in terms of energy utilization efficiency, while produced more milk. To promote low carbon development, more research with lactating Holstein dairy cows from different geographical locations, physiological stages, production systems in China is needed to establish regional or national GHG inventories as well as develop mitigation approaches to dairy production regimes.

## Supplementary Information

**Additional file 1: Table S1.** Carbon dioxide emissions of lactating Holstein dairy cows with different milk yields. **Table S2.** Carbon dioxide emissions of lactating Holstein dairy cows with different milk fat yields. **Table S3.** Carbon dioxide emissions of lactating Holstein dairy cows with different milk protein yields. **Table S4.** Carbon dioxide emissions of lactating Holstein dairy cows with different total milk solids yields.

## Data Availability

All data involved in this study are included in this article and its supplementary files.

## Abbreviations

ADF: Acid detergent fiber
Ca: Calcium
CH_4_: Methane
CO_2_: Carbon dioxide
CP: Crude protein
DM: Dry matter
DMI: Dry matter intake
ECM: Energy-corrected milk
EE: Ether extract
FAP: Fourth and above parity
FCM: Fat-corrected milk
GE: Gross energy
GHG: Greenhouse gas
HMFY: High milk fat yield
HMPY: High milk protein yield
HTMSY: High total milk solids yield
HMW: High metabolic weight
HMY: High milk yield
LMFY: Low milk fat yield
LMPY: Low milk protein yield
LTMSY: Low total milk solids yield
LMW: Low metabolic weight
LMY: Low milk yield
MMFY: Medium milk fat yield
MTMSY: Medium total milk solids yield
MMPY: Medium milk protein yield
MMW: Medium metabolic weight
MMY: Medium milk yield
MW: Metabolic weight
NE_L_: Net energy for lactation
NDF: Neutral detergent fiber
OM: Organic matter
P: Phosphorus
SP: Second parity
TMR: Total mixed rations
TP: Third parity
