# Supplementary material for: Characterization and mitigation option of greenhouse gas emissions from lactating Holstein dairy cows in East China
Source: J Anim Sci Biotechnol. 2022 Jun 30;13:88. doi: 10.1186/s40104-022-00721-3 (PMC9264640; doi:10.1186/s40104-022-00721-3)
Supplement: Supplementary file 1 — Additional file 1: Table S1. Carbon dioxide emissions of lactating Holstein dairy cows with different milk yields. Table S2. Carbon dioxide emissions of lactating Holstein dairy cows with different milk fat yields. Table S3. Carbon dioxide emissions of lactating Holstein dairy cows with different milk protein yields. Table S4. Carbon dioxide emissions of lactating Holstein dairy cows with different total milk solids yields. [file 40104_2022_721_MOESM1_ESM.docx]

Table S1. Carbon dioxide emissions of lactating Holstein dairy cows with different milk yields^1^

| Items^2^ | LMY | MMY | HMY | SEM | *P*-value |
| --- | --- | --- | --- | --- | --- |
| Total CO_2_, g/d | 18033^b^ | 19364^a^ | 20048^a^ | 20.28 | 0.042 |
| Total CO_2_/MW, g/kg | 131.6^b^ | 143.7^a^ | 146.7^a^ | 3.78 | 0.046 |
| Total CO_2_/ECM, g/kg | 624.9^a^ | 521.5^b^ | 457.9^c^ | 9.23 | 0.032 |

^1^LMY: low milk yield (< 34.7, *n* = 30); MMY, medium milk yield (34.7－41.5, *n* = 49); HMY, high milk yield (> 41.5, *n* = 32); *n*, number of observations in the data set.

^2^Total CO_2_, total CO_2_ production (g/d) = CH_4(CO2-eq)_ (g/d) + CO_2_ production (g/d). MW: metabolic weight (kg). ECM, Energy-corrected milk (kg/d) = milk yield (kg/d) × [(38.3 × fat (%) × 10 + 24.2 × protein (%) × 10 + 16.54 × lactose (%) × 10 + 20.7) ÷ 3140].

^a-c^Means in the same row with different superscripts are significantly different (*P* < 0.05)

Table S2. Carbon dioxide emissions of lactating Holstein dairy cows with different milk fat yields^1^

| Items^2^ | LMFY | MMFY | HMFY | SEM | *P*-value |
| --- | --- | --- | --- | --- | --- |
| Total CO_2_, g/d | 17884^b^ | 19751^a^ | 20132^a^ | 19.64 | 0.047 |
| Total CO_2_/MW, g/kg | 131.4^b^ | 145.1^a^ | 148.7^a^ | 3.87 | 0.036 |
| Total CO_2_/ECM, g/kg | 600.2^a^ | 524.1^b^ | 453.5^c^ | 7.75 | 0.029 |

^1^LMFY: low milk fat yield (< 1278, *n* = 39); MMFY: medium milk fat yield (1278－1550, *n* = 41); HMFY: high milk fat yield (> 1550, *n* = 31); *n*, number of observations in the data set.

^2^Total CO_2_, total CO_2_ production (g/d) = CH_4(CO2-eq)_ (g/d) + CO_2_ production (g/d). MW: metabolic weight (kg). ECM, Energy-corrected milk (kg/d) = milk yield (kg/d) × [(38.3 × fat (%) × 10 + 24.2 × protein (%) × 10 + 16.54 × lactose (%) × 10 + 20.7) ÷ 3140].

^a-c^Means in the same row with different superscripts are significantly different (*P* < 0.05)

Table S3. Carbon dioxide emissions of lactating Holstein dairy cows with different milk protein yields^1^

| Items^2^ | LMPY | MMPY | HMPY | SEM | *P*-value |
| --- | --- | --- | --- | --- | --- |
| Total CO_2_, g/d | 18001^b^ | 19431^a^ | 20095^a^ | 19.02 | 0.034 |
| Total CO_2_/MW, g/kg | 131.3^b^ | 145.0^a^ | 146.9^a^ | 3.67 | 0.043 |
| Total CO_2_/ECM, g/kg | 612.2^a^ | 524.4^b^ | 461.5^c^ | 5.98 | 0.039 |

^1^LMPY: low milk protein yield (< 1130, *n* = 35); MMPY: medium milk protein yield (1130－1364, *n* = 39); HMPY: high milk protein yield (> 1364, *n* = 37); *n*, number of observations in the data set.

^2^Total CO_2_, total CO_2_ production (g/d) = CH_4(CO2-eq)_ (g/d) + CO_2_ production (g/d). MW: metabolic weight (kg). ECM, Energy-corrected milk (kg/d) = milk yield (kg/d) × [(38.3 × fat (%) × 10 + 24.2 × protein (%) × 10 + 16.54 × lactose (%) × 10 + 20.7) ÷ 3140].

^a-c^Means in the same row with different superscripts are significantly different (*P* < 0.05)

Table S4. Carbon dioxide emissions of lactating Holstein dairy cows with different total milk solids yields^1^

| Items^2^ | LTMSY | MTMSY | HTMSY | SEM | *P*-value |
| --- | --- | --- | --- | --- | --- |
| Total CO_2_, g/d | 17924^b^ | 19486^a^ | 19998^a^ | 18.3 | 0.046 |
| Total CO_2_/MW, g/kg | 130.8^b^ | 144.9^a^ | 146.7^a^ | 6.70 | 0.045 |
| Total CO_2_/ECM, g/kg | 614.3^a^ | 530.0^b^ | 461.9^c^ | 7.41 | 0.038 |

^1^LTMSY: low total milk solids yield (< 4325, *n* = 33); MTMSY: medium total milk solids yield (4325－5115, *n* = 39); HTMSY: high total milk solids yield (> 5115, *n* = 39); *n*, number of observations in the data set.

^2^Total CO_2_, total CO_2_ production (g/d) = CH_4(CO2-eq)_ (g/d) + CO_2_ production (g/d). MW: metabolic weight (kg). ECM, Energy-corrected milk (kg/d) = milk yield (kg/d) × [(38.3 × fat (%) × 10 + 24.2 × protein (%) × 10 + 16.54 × lactose (%) × 10 + 20.7) ÷ 3140].

^a-c^Means in the same row with different superscripts are significantly different (*P* < 0.05)
